# The Effectiveness of Psychological Treatment for Anorexia Nervosa in Adolescents: A Critical Review of Randomized Controlled Trials

**DOI:** 10.1002/eat.70031

**Published:** 2026-01-30

**Authors:** Renée A. Broersma, Moniek A. J. Zeegers, Jesse Roest, Ramon J. L. Lindauer, Fabienne Harteveld, Peer van der Helm, James Lock, Mark Assink

**Affiliations:** ^1^ Child Development and Education University of Amsterdam Amsterdam the Netherlands; ^2^ Levvel Amsterdam the Netherlands; ^3^ Department of Child and Adolescent Psychiatry, Amsterdam UMC University of Amsterdam Amsterdam the Netherlands; ^4^ Faculty of Social Work and Applied Psychology Leiden University of Applied Sciences Leiden the Netherlands; ^5^ Department of Psychiatry and Behavioral Sciences Stanford University School of Medicine Stanford California USA

**Keywords:** adolescents, anorexia nervosa, family therapy, inpatient treatment, psychological treatment, randomized controlled trials, review

## Abstract

**Objective:**

Although international treatment guidelines for eating disorders recommend varying psychological approaches for adolescents with anorexia nervosa (AN), most existing reviews have combined adolescent and adult samples, leaving the overall evidence base for this population poorly defined. This systematic review is the first to synthesize randomized controlled trials (RCTs) of psychological treatments for adolescents with AN across outpatient, inpatient, and day‐patient settings.

**Method:**

A systematic search of databases (e.g., PubMed, APA PsycINFO) identified 22 relevant studies through June 2025. Eligible studies focused on adolescents (ages 8–18 years) with AN undergoing psychological treatment.

**Results:**

Evidence from the nine RCTs consistently indicates that family therapy produces significant positive effects on somatic parameters (e.g., weight) and short‐term eating‐disorder symptoms. In four, mostly underpowered comparative RCTs, outpatient family therapy showed small‐to‐moderate effect sizes over individual therapy, though these were restricted to outcomes related to medical recovery. For inpatient care, a limited evidence base suggests that shorter inpatient treatment followed by outpatient care may achieve outcomes comparable to extended hospitalization. Psychological modules delivered during inpatient treatment (e.g., Cognitive Remediation Therapy, CBT‐Insomnia) showed some improvements confined to narrow symptom domains but did not translate into broader recovery.

**Conclusion:**

The evidence base remains limited, with few RCTs in adolescents with AN and a geographically narrow, demographically homogeneous sample, predominantly girls from Western settings. Family therapy is supported as the first‐line treatment, yet substantial uncertainties persist. Larger and more inclusive RCTs are needed to clarify mechanisms of change, long‐term outcomes, and the effectiveness of individual‐focused approaches.

## Summary

- Evidence from randomized controlled trials supports family therapy as a first‐line treatment for adolescents with anorexia nervosa.
- Compared with individual therapy, family therapy shows stronger physical recovery outcomes (e.g., weight restoration and reduced rehospitalization), with no consistent advantage for psychological symptoms (e.g., eating‐disorder cognitions or emotional distress).
- Shorter inpatient treatment followed by outpatient care yields outcomes comparable to extended hospitalization, suggesting potential for more efficient care models.
- Major evidence gaps remain regarding long‐term outcomes and mechanisms of psychological change.
- Large‐scale RCTs are needed to clarify change processes, evaluate individual‐focused protocols, and determine how to tailor treatment to patient characteristics while maintaining family involvement.

## 1 Introduction

Anorexia nervosa (AN) is a severe, often chronic, and potentially life‐threatening mental illness characterized by an intense fear of weight gain and restrictive eating behaviors, resulting in significant weight loss and malnutrition (American Psychiatric Association [APA] 2013). AN affects 1%–2% of women and 0.2%–0.4% of men and has a mean duration of 5–7 years (Keski‐Rahkonen and Mustelin 2016). AN causes severe health problems like cardiovascular issues, osteoporosis, depression, and a higher risk of death from malnutrition or suicide (Arcelus et al. 2011; Westmoreland et al. 2016). It also profoundly impacts family members and caregivers, who report stress levels comparable to those of caregivers of children with life‐threatening illnesses, such as leukemia (Carmassi et al. 2021; Irish et al. 2024; Timko et al. 2023). In addition, health professionals treating individuals with AN often face burnout and emotional exhaustion (Hage et al. 2021; Johns et al. 2019). AN is expensive to treat, with frequent hospitalizations making it one of the costliest psychiatric disorders (Simon et al. 2005; Stuhldreher et al. 2012). Thus, despite its low prevalence, the wide‐reaching impact of AN on individuals, families, healthcare systems, and communities is undeniably significant.

AN most often emerges during adolescence, with peak onset between ages 14 and 19, a period marked by hormonal changes, identity development, and heightened sensitivity to social influences (Solmi et al. 2021). The disorder arises from a complex interplay of genetic (Bulik et al. 2007), neurobiological, psychological, and sociocultural factors, with heritability estimated at 48%–74% (Yilmaz et al. 2015). Although short‐term mortality is lower than in adults, adolescents frequently experience severe and lasting medical complications, including growth delay, osteoporosis, and structural and functional brain changes (Hudson et al. 2012; Misra and Klibanski 2011; Seitz et al. 2016). Early‐onset AN is further linked to poorer long‐term outcomes, greater psychiatric comorbidity (e.g., personality disorders, autism, social anxiety disorder), and increased life difficulties (Grilo and Udo 2021). These risks highlight the importance of early recognition and intervention to improve prognosis (Katzman 2005; Treasure and Russell 2011).

In the present study, we aimed to review the effectiveness of treatments for adolescents with AN. Global treatment guidelines for AN recommend distinct treatment approaches for adults and adolescents, with the most notable distinction being an individual‐based approach for adults and a family‐based approach for adolescents (e.g., Crone et al. 2023; Couturier et al. 2020; GGZ Zorgstandaarden 2025; Heruc et al. 2020; NICE 2024). To date, over 20 systematic reviews and meta‐analyses have examined AN treatment, primarily in adult or mixed‐age samples, or have explored age‐related differences in treatment effects through moderator analyses (see Monteleone et al. 2022, for an overview). Only a limited number of studies (three to our knowledge) have systematically synthesized empirical findings on the effectiveness of specific AN treatment approaches for adolescents (Austin et al. 2025; Couturier et al. 2013; Wergeland et al. 2025). Notably, the available reviews and meta‐analyses on youth with AN have focused primarily on a subset of treatment recommendations outlined in current international guidelines, such as outpatient family‐based treatments (FBTs). To date, previous reviews have not assessed the effectiveness of treatments for adolescents across different care settings, including outpatient, inpatient, and day‐patient care.

To address this gap, we conducted a comprehensive review of the evidence supporting different AN treatments recommended in global guidelines, focusing exclusively on adolescents. Our objective was to evaluate the extent to which current global treatment guidelines for youth with AN are supported by empirical evidence and to identify research gaps that emerge when considering these guidelines.

### 1.1 Unique Challenges of Treating AN in Adolescents

An important reason to consider treatment effects separately for adolescents with AN is the presence of unique treatment dynamics. First, given the heightened risks associated with severe malnutrition in adolescents, healthcare providers often prioritize physical stabilization and weight restoration to prevent long‐term complications and support healthy growth. However, this urgent focus on physical health may create tension with the adolescent's readiness for psychological treatment, complicating the integration of physical and psychological care and potentially impacting the overall effectiveness of the treatment approach (Westwood and Kendal 2012).

Second, adolescents must navigate complex family dynamics, balancing their dependence on caregivers with a growing need for autonomy. This is particularly challenging in AN because it is an ego‐syntonic disorder: many eating‐disorder thoughts and behaviors, such as restricting food, avoiding weight gain, or feeling “in control” when eating less, are experienced as reasonable or even necessary. This makes it difficult for adolescents to recognize these patterns as harmful, which further complicates change during a developmental stage centered on increasing independence (Gregertsen et al. 2017). Paradoxically, during AN treatment, autonomy restrictions such as force‐feeding and hospitalization are more common for adolescents than for adults (Carney et al. 2008; Elzakkers et al. 2014). Adolescents with AN are more likely to face physical restraints and compulsory nutritional interventions due to their perceived lack of competency to refuse treatment and the urgent need to prevent severe health deterioration or death (Elzakkers et al. 2014). Also, in many jurisdictions, adolescents do not have the legal autonomy to refuse medical treatment or hospitalization, especially if they are deemed to be at risk of serious harm due to their eating disorder. While often necessary to protect physical health, these measures can exacerbate feelings of powerlessness and resistance, creating additional trauma and challenges in building trust and fostering a collaborative therapeutic relationship. Research suggests that these mechanisms are not limited to adolescence but often continue into adulthood, where difficulties with autonomy, identity, and comorbid mental health problems can also shape the course of AN and its response to treatment (e.g., Burke et al. 2024; Hope et al. 2013; Westmoreland et al. 2024).

### 1.2 Global Treatment Guidelines for AN

Global treatment guidelines for eating disorders are developed through a process involving evidence synthesis, expert input, and consideration of real‐world clinical challenges. Treatment guidelines for AN consistently emphasize that psychological interventions for adolescents with AN should differ from those designed for adults (e.g., Crone et al. 2023; Couturier et al. 2020; GGZ Zorgstandaarden 2025; Heruc et al. 2020; NICE 2024). Treatment recommendations for adults focus on fostering greater collaboration with the individual, encouraging them to take an active role in their recovery. Therapy protocols typically recommended as first‐line treatments for adults include individual therapies, such as eating disorder‐focused cognitive behavioral therapy (CBT‐ED), Maudsley model of Anorexia Nervosa Treatment for Adults (MANTRA), or Specialist Supportive Clinical Management (SSCM) (Fairburn et al. 2003; Jordan et al. 2020; Zipfel et al. 2015).

In contrast, treatment guidelines for children and adolescents prioritize an outpatient family‐based approach. This shift is driven by evidence from RCTs showing that involving families improves outcomes (e.g., Russell et al. 1987; Robin et al. 1999). Historically, however, treatment often excluded families, as early models viewed them as part of the problem rather than the solution (e.g., Minuchin et al. 1978). The family‐based approach is centered around active family participation, with a focus on meal planning, weight restoration, and parental support.

While family‐based approaches are the primary recommendation for children and young people with AN, individual therapies such as CBT‐E or adolescent‐focused psychotherapy should be considered when family therapy is ineffective (e.g., NICE 2024). There is increasing recognition that particular adult treatments can be adapted across the adolescent‐adult age divide, such as MANTRA for adolescents (e.g., Wittek et al. 2021). Below we summarize the recommended types of psychological treatment for adolescents with AN that are reported in international treatment guidelines from Australia/New Zealand, Canada, Germany, the Netherlands, the United Kingdom, and the United States (Crone et al. 2023; Couturier et al. 2020; GGZ Zorgstandaarden 2025; Heruc et al. 2020; NICE 2024; Resmark et al. 2019).

#### 1.2.1 Outpatient Treatment for Adolescents With AN

##### 1.2.1.1 Family Treatment Programs for AN

For children and adolescents with AN, treatment guidelines consistently recommend eating disorder‐focused family therapy in an outpatient setting as the first‐line intervention. This form of family therapy has been referred to by various names, including the Maudsley approach, the Maudsley Model of Family Therapy, and FBT. The term Maudsley originates from its development at the Maudsley Hospital in London, where the early research and clinical trials of this family‐based intervention were conducted (Eisler et al. 2015). However, these terms can be ambiguous, as they are sometimes used to refer specifically to treatment manuals. To ensure consistency and clarity, this article will use the term eating disorder‐focused family therapy as a broad umbrella term. Within this framework, we will discuss adaptations of the approach for AN, encompassing both single‐family and multi‐family formats. Further, terms such as FBT or Behavioral Family Systems Therapy for AN (BFST) will be used exclusively to refer to studies that apply specific manualized versions of this treatment. For a detailed overview of the history of eating disorder‐focused family therapies, we refer readers to Eisler et al. (2015).

Among the various eating disorder‐focused family therapies that have been studied, FBT (Lock and Le Grange 2005, 2012) has received the most attention and is the focus of multiple effectiveness studies and RCTs (Lock 2019). FBT for adolescent AN was first manualized in 2001 (Lock and Le Grange 2001), which facilitated its subsequent use in studies and its dissemination and implementation. FBT directly involves the parents in the therapeutic process, emphasizing collaboration among family members to support the adolescent's recovery. The core principle of FBT is to support and empower parents in guiding their child's eating habits, ensuring consistent and structured nutrition until the adolescent develops the capacity to make healthy, independent decisions.

The therapy is structured in three phases (Lock and Le Grange 2012): first, parents assume responsibility for their child's eating and weight restoration, providing close supervision of meals to ensure adequate nutritional intake. The second phase gradually shifts responsibility for eating back to the adolescent as their weight stabilizes. The final phase addresses broader family dynamics and the adolescent's developmental issues, promoting autonomy and healthy family relationships. FBT typically consists of approximately 20 sessions over a period of 6–12 months. Two additional eating disorder‐focused family therapies with similar approaches have also been evaluated for their effectiveness: BFST (Robin et al. 1999) and Family Therapy for AN (FT‐AN; also described as “conjoint family therapy”; Eisler et al. 2000, 2007).

Multifamily Therapy (MFT) was developed in the 1990s to enhance single‐family therapy by introducing a more collaborative and intensive format (Dare and Eisler 2000; Scholz and Asen 2001). In MFT, 5–7 families participate in structured sessions that combine group therapy, psychoeducation, creative exercises, and practical guidance, such as multifamily meals. Adaptations like MFT‐AN (Eisler et al. 2016; Simic et al. 2022) are rooted in single‐family therapy models (e.g., FBT, FT‐AN). Programs vary in length and intensity; for example, Eisler et al. (2016) evaluated an intensive four‐day program followed by six 1‐day sessions across 9 months. MFT employs a diverse range of techniques, including group therapy, psychoeducation, creative exercises, and separate sessions for parents and adolescents (Eisler 2005; Le Grange et al. 2020). It also includes practical guidance on managing mealtimes, such as multifamily meals and discussions. In clinical practice, it is often combined with individual family treatment.

An alternative to an eating disorder‐focused family therapy is manualized systemic family therapy (SyFT; Pote et al. 2003), which focuses on improving overall family dynamics rather than targeting eating‐specific behaviors. SyFT is structured into three phases. First, the focus is on building a trusting therapeutic relationship and gathering detailed information about the family's current interaction and communication styles, identifying both strengths and challenges (Agras et al. 2014; Pote et al. 2003). Once these patterns are understood, phase two begins, in which the family is encouraged to critically evaluate and address the interactions they find problematic and wish to improve. The final phase involves supporting the family as they experiment with and implement new interaction and communication strategies. SyFT typically involves 10–20 sessions delivered over a period of 6–12 months.

##### 1.2.1.2 Individual Treatment Programs for AN

When family treatment is not feasible or presumed ineffective for children or young people with AN, global treatment guidelines recommend an individual approach to therapy (e.g., Crone et al. 2023; GGZ Zorgstandaarden 2025; Heruc et al. 2020; NICE 2024). The most studied individual therapy for adolescents with AN is Adolescent‐Focused Therapy for Anorexia Nervosa (AFT‐AN), also known as Ego‐Oriented Individual Therapy (EOIT; Robin et al. 1999). This therapy was developed as an alternative to FBT and BFST (Fitzpatrick et al. 2010; Le Grange et al. 2016; Robin et al. 1999). AFT‐AN is rooted in psychodynamic principles, focusing on the adolescent's autonomy, identity development, and emotional regulation, which are often disrupted by the eating disorder (Fitzpatrick et al. 2010; Lock et al. 2010). AFT‐AN emphasizes the adolescent's role in understanding and addressing their eating behaviors while exploring underlying psychological factors, such as perfectionism or relational issues (Fitzpatrick et al. 2010). The treatment typically involves 24–32 weekly sessions over 6–8 months, offering a structured yet flexible approach.

Second, for adults with AN, CBT and particularly its enhanced form (CBT‐E) is the most extensively studied and commonly used treatment (Fairburn et al. 2003; Fairburn 2008; Öst et al. 2024). CBT‐E is a transdiagnostic, evidence‐based treatment developed by Fairburn (2008) to address the core mechanisms maintaining eating disorders, such as overvaluation of shape and weight, dietary restraint, and perfectionism. The therapy is structured into four stages: early stabilization of eating patterns, progress review, addressing maintaining factors, and relapse prevention. It is typically delivered in 20 sessions over 20 weeks for individuals not significantly underweight and up to 40 sessions over 40 weeks for those requiring weight restoration, such as in AN (Fairburn 2008; Byrne et al. 2011). For adolescents, CBT‐E has been adapted to include specific protocols such as one that incorporates structured family involvement (Dalle Grave and Calugi 2024).

The application of CBT‐E in adolescents has been less studied than in adults, though evidence is growing (e.g., Le Grange et al. 2020; Dalle Grave et al. 2016). A central challenge is that CBT‐E requires the individual to take ownership of the recovery process, which may be difficult for adolescents given their developmental stage and the severe medical consequences of AN in this age group (Hudson et al. 2012; Le Grange et al. 2020; Misra and Klibanski 2011; Nicholls and Barrett 2015). Nevertheless, recent non‐randomized evidence suggests CBT‐E may achieve outcomes comparable to FBT, with families more often selecting CBT‐E for older adolescents with longer illness duration and greater psychosocial impairment (Le Grange et al. 2020).

### 1.3 Inpatient Treatment for Adolescents With AN

#### 1.3.1 Inpatient Treatment Programs for AN

When individuals with AN (adults or adolescents) require more support than outpatient family or individual programs can offer, international treatment guidelines recommend inpatient treatment programs for AN that can provide more intensive and comprehensive care (e.g., Crone et al. 2023; GGZ Zorgstandaarden 2025; Heruc et al. 2020; NICE 2024). These programs are typically offered in hospitals or specialized treatment centers and involve a multidisciplinary approach. Key components of inpatient care typically include medical stabilization, nutritional counseling, therapeutic and behavioral interventions, and psychiatric care (e.g., use of medication).

Inpatient treatment is sometimes criticized for high costs and overemphasizing medical stabilization at the expense of addressing underlying psychological and social factors, increasing the risk of relapse (Guarda et al. 2007; Herpertz‐Dahlmann et al. 2014; Nyttingnes et al. 2018; Toulany et al. 2015). Prolonged inpatient care can also strain family relationships by creating physical separation and limiting opportunities for families to play an active role in the recovery process. This has led a few studies to examine the effects of inpatient treatment programs compared to other care settings, such as day care programs (Gowers et al. 2007; Haas et al. 2022; Madden et al. 2015). In the present review, we evaluate the evidence for the overall effectiveness of inpatient treatment programs for adolescents with AN, focusing on studies that assess the full course of treatment and compare outcomes to other settings such as day care and outpatient care.

#### 1.3.2 Psychological Treatment Modules Within Inpatient Programs for Adolescents With AN

In recent years, an increasing number of studies have explored the effectiveness of specific psychological modules embedded within broader inpatient treatment programs for adolescents with AN. Rather than evaluating inpatient care as a uniform intervention, these studies focus on isolating and testing the added value of particular group‐based therapies or adjunctive modules implemented alongside standard care. As mentioned above, standard inpatient programs typically include medical stabilization, nutritional support, behavioral interventions, and individual or group psychotherapy. However, researchers have begun to question whether tailoring these programs with targeted psychological components can further enhance treatment outcomes.

This growing line of inquiry reflects a broader shift toward more modular, mechanism‐focused approaches that address specific psychological or neurocognitive features of AN – such as cognitive rigidity, low self‐esteem, or sleep disturbances – that may otherwise limit recovery. In the present review, we evaluate the evidence for these adjunctive interventions by synthesizing findings from studies that directly compared standard inpatient care with and without the addition of specialized psychological modules. This allows us to assess the potential contribution of such interventions to the overall effectiveness of inpatient treatment for adolescents with AN.

### 1.4 The Present Study

This study addresses the need for a comprehensive overview of the existing evidence (RCTs) on psychological treatments for adolescents with AN across various settings. Previous reviews have not systematically considered the evidence for the diverse inpatient and outpatient recommendations typically presented in global guidelines for adolescent AN treatment (e.g., Crone et al. 2023; Couturier et al. 2020; Heruc et al. 2020; NICE 2024). Overall, these guidelines generally recommend (a) eating disorder‐focused family therapy, (b) that additional or standalone individual psychological treatment should be considered in cases where family treatment is insufficient, and (c) that treatment should be intensified with day patient or inpatient treatment when outpatient care alone is insufficient. To comprehensively evaluate the available evidence for the different treatment approaches and the settings in which they are offered to adolescents with AN, we pose five research questions.

Research Question 1 “What is the effectiveness of outpatient eating disorder‐focused family therapy for adolescents with AN when compared to outpatient individual psychological therapy?” Current treatment guidelines for adolescents with AN consistently recommend family‐based approaches as the first‐line intervention. Previous studies have suggested that family therapy may be superior to individual therapy on key medical and somatic outcomes (e.g., Couturier et al. 2013; Lock et al. 2010). We therefore hypothesized that FBT would yield superior treatment outcomes compared with individual psychological therapy in this population.

Research Question 2 “What is the effectiveness of outpatient single‐family therapies, such as FBT, FT‐AN and BFST, for adolescents with AN when compared to other family‐based approaches, such as MFT and SyFT?” We examined differences between family treatment approaches by comparing the effects of single‐family therapies for adolescent AN with those of other family‐based interventions, with no a priori hypotheses about where potential differences in treatment outcomes may lie.

Research Question 3 “What is the effectiveness of outpatient individual psychological therapy on recovery and long‐term outcomes for adolescents with AN?” Unlike Research Question 1, for which we formulated an a priori expectation about the superiority of FBT over individual therapy, this question aims solely to synthesize the evidence on outpatient individual therapies for adolescents with AN, without a priori hypotheses regarding potential treatment outcomes.

Research Question 4 “What is the effectiveness of inpatient treatment compared to outpatient or day patient treatment for adolescents with AN?” Given that inpatient treatment is costly and highly intrusive for adolescents, this question synthesizes the available evidence on the impact of comprehensive inpatient treatment programs across a range of outcomes compared with outpatient or day‐patient care, without a priori hypotheses about potential treatment differences.

Research Question 5 “What is the effectiveness of psychological treatment modules embedded within inpatient programs for adolescents with AN?” Inpatient treatment programs often include different therapeutic modules aimed at enhancing recovery. It is unclear whether there is convincing evidence for the effectiveness of specific modules within inpatient treatment and whether adding these targeted interventions enhances clinical outcomes. Therefore, this question sought to evaluate the impact of group therapy modules on both immediate recovery and long‐term outcomes, without a priori hypotheses.

Recognizing the limited number of available RCTs, we first assessed the feasibility of calculating an overall effect size for each research question upon completing the literature search.

## 2 Method

### 2.1 Study Search and Selection

**FIGURE 1:**
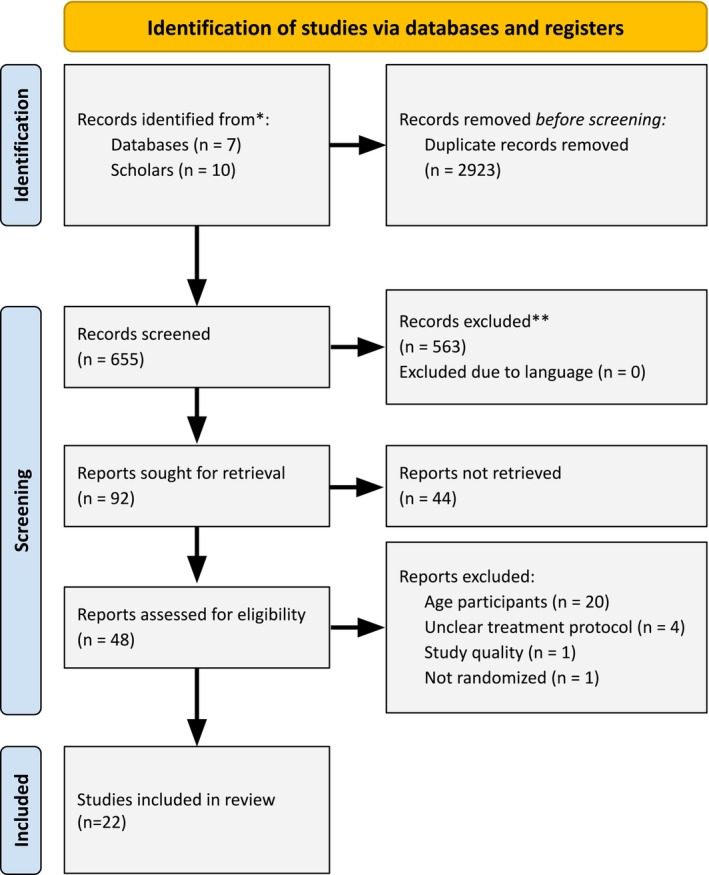
PRISMA flow chart of the search strategy and identification of studies.

This systematic review was conducted in accordance with the *Preferred Reporting Items for Systematic Reviews and Meta‐Analyses* (PRISMA) 2020 guidelines (Page et al. 2021) (registration PROSPERO CRD42023487988). To identify eligible studies for inclusion, three complementary search strategies were used. First, seven electronic databases were initially searched between April 2024 and June 2025, with a final search conducted on June 16, 2025, for articles, book chapters, dissertations, and reports on AN treatment in adolescents: Web of Science, APA PsycINFO, Google Scholar, Pubmed, Embase, Cochrane Library, and ERIC. The following umbrella keywords were used: “anorexia,” “therap*,” “intervention,” “treatment,” “adolesc*,” “youth*,” “juvenile*,” “experiment,” “control group,” “control condition,” “comparison condition,” and “randomized controlled trial,” which were supplemented with conjugations and synonyms. Appendix S1 presents an explanation of the search strategy. The search identified 645 unique potentially relevant manuscripts, of which we first screened titles and abstracts. Next, full article texts of potentially relevant studies were read and evaluated against the inclusion criteria. To complement this electronic search, reference lists of previous meta‐analyses (e.g., Austin et al. 2025; Couturier et al. 2013; Monteleone et al. 2022) were screened. Finally, 10 scholars in the eating disorder field were contacted in attempting to locate unpublished studies. We received 10 studies, of which 2 met the inclusion criteria. The PRISMA flow diagram of the study selection process is provided in Figure 1.

### 2.2 Inclusion Criteria

Studies were included based on the following criteria:

#### 2.2.1 Study Design

Eligible for inclusion were RCTs, cluster‐RCTs, and cross‐over trials. As for language and publication year of studies, we did not impose any restriction.

#### 2.2.2 Participant Characteristics

Studies had to sample adolescents with a mean sample age of < 19 years. We applied this criterion to ensure that participants had received treatment within the adolescent care system rather than adult services. Because many studies included participants up to 23 years of age (e.g., Ball and Mitchell 2004) but reported a mean age around 18 years, we included such studies as long as the mean sample age did not exceed 19 years. Adolescents were required to have a primary clinical diagnosis of AN, either the restricting or purging subtype, based on the diagnostic criteria applied in the respective study, which could include DSM‐5 criteria (Crone et al. 2023), ICD criteria (e.g., ICD‐10; WHO 1992), or clinicians' own diagnostic assessments. Primary study participants could have AN of any severity, including chronic AN, and participants could have psychiatric comorbidities. The sampled adolescents could have received the studied intervention in various care settings, including inpatient, day patient, or outpatient settings. The participants may have joined the trial either at treatment initiation or after the trial had started, for instance, following hospital discharge or once weight stabilization (as defined by the primary study) was achieved.

#### 2.2.3 Interventions

Across research questions, different interventions were synthesized.

Research Question 1 Trials examining interventions that involved family members were included. Most of these interventions were based on FBT (Lock et al. 2010) and one was based on BFST (Robin et al. 1999). Participants in the control condition of these trials had to be offered individual therapy, such as CBT, psychodynamic therapy (e.g., affect‐focused therapy, EOIT), or supportive therapy. Studies with a treatment‐as‐usual (TAU) control condition were excluded (e.g., Godart et al. 2012), because the nature of TAU may differ substantially across studies, which hampers drawing firm conclusions about the effectiveness of the intervention that is offered in the experimental condition. Notably, TAU may be (partly) based on parental support sessions, which confounds family intervention effects and may obscure the unique impact of structured family therapy.

Research Question 2 Trials examining interventions that involved a conjoint single‐family therapy (e.g., FBT, FT‐AN, and BFST) were included. The control conditions in these trials involved family‐based interventions that employed different approaches, such as those not explicitly focused on modifying eating behaviors or those treating parents separately from the child (e.g., SyFT or parent‐focused therapy).

Research Question 3 In addition to Research Question 1, we aimed to examine whether any individual psychological interventions for AN have been systematically evaluated. Included were trials that examined individual psychological interventions for AN, such as CBT and its derivatives, psychodynamic therapy (AFT), metacognitive training (MCT), interpersonal therapy, supportive therapy, and play therapy. Given the limited number of available studies evaluating effects of individual therapy for adolescents with AN, we chose not to exclude studies based on the nature of the control condition, which enabled us to provide a systematic description of the available evidence. Consequently, the included studies had control conditions that involved interventions provided either as standalone treatments (monotherapies) or as part of standard care (TAU), sometimes in combination with other therapies. Also, we excluded studies in which family therapy was used as a control condition, as these studies had already been considered in the analysis of Research Question 1.

Research Question 4 Trials examining a multimodal “inpatient” program with 24‐h care were included. These programs were focused on medical stabilization, nutritional counseling, and the provision of therapeutic and behavioral interventions. Given the limited number of available studies evaluating effects of comprehensive inpatient programs for AN adolescents, we did not exclude studies based on the nature of the control condition. Consequently, the included studies featured control conditions encompassing various treatment settings, such as standard outpatient care and day treatment programs.

Research Question 5 Included were all trials that compared adolescents receiving a psychological intervention alongside standard inpatient care in the experimental condition with adolescents receiving only standard inpatient care in the control condition. The psychological interventions in the experimental condition could involve individual or group‐based approaches.

### 2.3 Data Analytic Strategy

For each research question, we evaluated whether pooling effect sizes would be appropriate. The number of eligible RCTs was small, ranging from two to seven per research question, and the studies varied substantially in design and content. Conjoint family therapies such as FBT were compared with conceptually distinct approaches, including MFT, SyFT, and parent‐focused formats, each defined by different therapeutic components. Comparator conditions also differed markedly, ranging from supportive counseling and psychodynamic therapy to CBT, while treatment duration spanned from nine sessions (Le Grange et al. 1992) to 25 sessions (Ball and Mitchell 2004; Lock et al. 2010). This level of clinical and methodological diversity undermines the validity of pooled estimates, and the small sample sizes precluded adequately powered moderator analyses.

Considering these limitations, we conducted a systematic review with structured narrative synthesis. Trial results were synthesized by systematically considering the intervention and comparator conditions, sample and treatment characteristics, outcome domains, dropout rates, and principal findings. This approach allows for a critical appraisal of the available evidence and a more meaningful comparison across studies. We qualitatively assessed the overall certainty of evidence for each outcome, considering study quality, sample size, consistency of findings, and precision of reported effects. For Research Questions 1 and 2, we additionally present tables with converted Hedges' *g* effect sizes and their 95% confidence intervals, as these questions compared two clearly defined active treatment arms (e.g., family‐based versus individual therapy, or single‐family vs. other family‐based therapy), which allowed for standardized estimation of between‐group effect sizes (Hedges' *g*).

Only outcomes directly related to adolescents with AN were included. Outcomes concerning parents, families, or relational dynamics were excluded. The extracted outcomes were grouped into four main domains: rehospitalization, remission, somatic/medical outcomes, and psychological outcomes:

*Hospitalization*. Hospitalization refers to the proportion of participants requiring medical or psychiatric inpatient admission during or after treatment.

*Remission*. Remission was defined as partial or full recovery from AN symptoms, typically operationalized through achieving a healthy weight (≥ 85%–95% of ideal body weight) and normalized eating‐disorder scores (e.g., EDE; Lock et al. 2010).

*Somatic/Medical outcomes*. These outcomes included weight restoration (e.g., BMI, %mBMI, weight gain), remission rates, hospitalization, and somatic functioning (e.g., menstrual function, Morgan‐Russell physical subscale).

*Psychological outcomes*. These outcomes included eating disorder psychopathology, assessed with validated self‐report or interview‐based instruments such as Eating Attitudes Test (EAT, Garner and Garfinkel 1979), Eating Disorder Examination‐Questionnaire (EDE‐Q; Fairburn and Cooper 1993), or the Eating Disorders Inventory (Garner 1991, 2004; Garner et al. 1983). Internalizing symptoms, including anxiety and depression, were assessed using standardized measures such as the Child Behavior Checklist (CBCL, Achenbach and Rescorla 2001), Youth Self‐Report (YSR; Achenbach and Rescorla 2001), or the Beck Depression Index (BDI; Beck et al. 1996).

## 3 Results

### 3.1 Research Question 1. Comparison of Outpatient Family Therapy and Individual Therapy

Four RCTs met the inclusion criteria to compare the effectiveness of outpatient, eating disorder‐focused family therapy with individual psychological therapy (Table 1). The findings, though methodologically diverse, consistently indicate that the primary added value of family therapy lies in promoting somatic and medical recovery, while its effects on psychological outcomes are largely comparable to those of individual therapy.

**TABLE 1:** Studies evaluating outpatient family therapy to individual therapy.

Study	Demographics	Family therapy	Compared to	*n* (intervention)	*n* (comparison)
Ball and Mitchell (2004)	Mean age = BFT: 17.58; CBT: 18.45 Age range = 13–23 years Female = 100% Race/ethnicity = NR SES = NR	BFST	CBT	9	9
Lock et al. (2010)	Mean age = FBT: 14.1 (SD = 1.7); AFT: 14.7 (SD = 1.5) Age range = 12–18 years Female = 91% Race/ethnicity = Asian 11%, Black 1%, Caucasian 76%, Hispanic 7%, ethnic minority 24% SES = Parent education in years: *M* = 16.7 (SD = 2.6)	FBT	AFT‐AN	61	60
Robin et al. (1999)	Mean age = BFST 14.9; EOIT 13.4 Age range = 11–20 years Female = 100% Race/ethnicity = White 94.6%, Middle Eastern 5.4% SES = (Hollingshead four‐factor index) BFST *M* = 45.7 (SD = 13.6); EOIT *M* = 47.9 (SD = 12.0)	BFST	EOIT	19	18
Russell et al. (1987)	Mean age = 16.6 years (SD = 1.7) Age range = ≤ 18 years Female = NR^a^ Race/ethnicity = NR SES = NR^a^	Family therapy^b^	Individual supportive therapy/counseling	10	11

Abbreviation: NR, not reported.

^a^ The study describes only totals that include subgroups not included in this review.

^b^ The family therapies in Russell et al. (1987) are a precursor of manualized family treatments (FBT).

Across the four randomized trials comparing outpatient family therapy to individual therapy for adolescents with AN, substantial variability was observed in study design, sample size, and outcome assessment (Table 2). The largest and most robust trial by Lock et al. (2010; *N* = 121) provided adequate power to detect moderate between‐group effects, whereas Ball and Mitchell (2004) enrolled only nine participants per arm, rendering them underpowered to detect small‐to‐moderate effects. Treatment format varied: Russell et al. (1987) examined outpatient family therapy as a maintenance strategy following inpatient weight restoration, Robin et al. (1999) tested BFST that blended family and cognitive‐behavioral strategies, and Lock et al. investigated manualized FBT. The individual‐therapy comparators ranged from supportive psychotherapy to ego‐oriented and CBT‐like approaches. Outcome domains were medical or somatic recovery measures (e.g., BMI, weight, menstrual function) and standardized psychological measures such as the EDE‐Q, EDI subscales, or depression scales (e.g., Beck Depression Index). One study reported on remission status (Lock et al. 2010) and two on hospitalization rates (Lock et al. 2010; Robin et al. 1999). Follow‐up periods ranged from end‐of‐treatment to 12 months.

**TABLE 2:** Detailed overview of outcome measures: eating disorder‐focused single‐family therapies versus individual therapy.

Study	Type of outcome (medical/somatic; psychological; remission; rehospitalization)	Outcome measure/Instrument	Hedges *g* ^a^, 95% CI posttest (PT) or follow‐up (F‐U)
Ball and Mitchell (2004)	Medical/somatic	BMI	−0.26, 95% CI [−1.19, 0.67] (PT) 0.16, 95% CI [−0.77, 1.08] (F‐U)
		Morgan Russell Assessment Schedule (composite)^b^	−0.13, 95% CI [−1.05, 0.80] (PT) −0.17, 95% CI [−1.09, 0.76] (F‐U)
	Psychological	EDE‐Q (composite)	0.09, 95% CI [−0.84, 1.01] (PT) 0.19, 95% CI [−0.74, 1.12] (F‐U)
		EDI‐2 Body dissatisfaction	−0.16, 95% CI [−1.09, 0.77] (PT) 0.20, 95% CI [−0.73, 1.13] (F‐U)
		EDI‐2 Perfectionism	−0.16, 95% CI [−1.09, 0.76] (PT) 0.03, 95% CI [−0.89, 0.95] (F‐U)
		EDI‐2 Interoceptive awareness	−0.22, 95% CI [−1.15, 0.71] (PT) 0.31, 95% CI [−0.62, 1.24] (F‐U)
		BDI	−0.63, 95% CI [−1.58, 0.31] (PT) −0.46, 95% CI [−1.40, 0.47] (F‐U)
		STAI	0.03, 95%, CI [−0.90, 0.95] (PT) 0.26, 95%, CI [−0.67, 1.18] (F‐U)
Lock et al. (2010)	Remission	Remission rate	0.54, 95% CI [0.15, 0.93] (PT) 0.64, 95% CI [0.22, 1.07] (F‐U 6 m) 0.71, 95% CI [0.29, 1.13] (F‐U 12 m)
	Rehospitalization	Number of hospitalizations	0.70, 95% CI [0.30, 1.10] (PT) 0.49, 95% CI [0.06, 0.91] (F‐U 6 m)
	Medical/somatic	BMI percentiles	0.40, 95% CI [0.01, 0.79] (PT) 0.10, 95% CI [−0.32, 0.52] (F‐U 6 m) 0.14, 95% CI [−0.27, 0.54] (F‐U 12 m)
	Psychological	EDE‐Q (composite)	0.44, 95% CI [0.05, 0.83] (PT) 0.21, 95% CI [−0.21, 0.63] (F‐U 6 m) 0.23, 95% CI [−0.18, 0.64] (F‐U 12 m)
Robin et al. (1999)	Rehospitalization	Number of hospitalizations	−0.77, 95% CI [−1.44, −0.10] (PT)
	Medical/somatic	BMI	1.22, 95% CI [0.51, 1.93] (PT) 1.01, 95% CI [0.31, 1.70] (F‐U)
	Psychological	EAT—self	0.48, 95% CI [−0.19, 1.16] (PT) 0.36, 95% CI [−0.31, 1.03] (F‐U)
		EAT—father	0.13, 95% CI [−0.59, 0.86] (PT) 0.01, 95% CI [−0.71, 0.74] (F‐U)
		EAT—mother	0.59, 95% CI [−0.08, 1.26] (PT) 0.03, 95% CI [−0.62, 0.68] (F‐U)
		EDI‐2 Perfectionism	0.25, 95% CI [−0.43, 0.92] (PT) 0.41, 95% CI [−0.27, 1.10] (F‐U)
		EDI‐2 Interoceptive awareness	0.44, 95% CI [−0.24, 1.12] (PT) 0.16, 95% CI [−0.51, 0.84] (F‐U)
		EDI‐2 Maturity Fears	−0.09, 95% CI [−0.77, 0.58] (PT) −0.37, 95% CI [−1.05, 0.31] (F‐U)
		EDI‐2 Ineffectiveness	−0.21, 95% CI [−0.88, 0.47] (PT) −0.22, 95% CI [−0.89, 0.46] (F‐U)
		EDI‐2 Interpersonal distrust	−0.32, 95% CI [−1.00, 0.36] (PT) −0.27, 95% CI [−0.95, 0.40] (F‐U)
		BDI	0.34, 95% CI [−0.33, 1.01] (PT) −0.19, 95% CI [−0.85, 0.48] (F‐U)
		CBCL (internalizing)—father	−0.23, 95% CI [−0.87, 0.42] (PT) −0.09, 95% CI [−0.73, 0.56] (F‐U)
		CBCL (internalizing)—mother	0.40, 95% CI [−0.25, 1.05] (PT) 0.08, 95% CI [−0.56, 0.73] (F‐U)
		YSR (internalizing)—teen	−0.67, 95% CI [−1.33, −0.01] (PT) −0.56, 95% CI [−1.22, 0.10] (F‐U)
Russell et al. (1987)	Medical/somatic	Weight	−0.19, 95% CI [−1.05, 0.66] (PT) 0.66, 95% CI [−0.22, 1.54] (F‐U)
		Morgan Russell Assessment Schedule—Nutritional status	2.05, 95% CI [1.00, 3.11] (F‐U)
		Morgan Russell Assessment Schedule—Menstrual function	1.00, 95% CI [0.09, 1.91] (F‐U)
	Psychological	Morgan Russell Assessment Schedule—Mental State	0.70, 95% CI [−0.18, 1.59] (F‐U)
		Morgan Russell Assessment Schedule—Psychosexual adjustment	0.98, 95% CI [0.07, 1.88] (F‐U)

Abbreviations: BDI, Beck Depression Inventory; EAT, Eating Attitudes Test; EDE‐Q, Eating Disorders Examination; EDI‐2, Eating Disorders Inventory; F‐U, follow‐up; PT, post‐test; STAI, State–Trait Anxiety Inventory.

^a^ Hedges'*g*, 0.20 = small, 0.50 = moderate, 0.80 = large, respectively (Cohen 1988).

^b^ The Morgan–Russell Assessment Schedule composite includes both medical and psychological subscales (Morgan and Hayward 1988).

Regarding the outcomes of the separate studies, most studies showed higher effect sizes for family therapy for core medical outcomes. Lock et al. (2010) reported significantly higher full‐remission rates for family therapy at 6‐ and 12‐month follow‐up (49% vs. 23%; Number Needed to Treat [NNT] ≈4) and lower hospitalization rates (15% vs. 37%). Robin et al. (1999) similarly found greater weight gain in the family therapy condition (mean BMI increase = 4.7 kg/m^2^) than in individual therapy (2.3 kg/m^2^). In Russell et al. (1987), family therapy was superior in maintaining weight post‐discharge among adolescents with early‐onset and shorter illness duration, with a mean gain of 3.4% compared to an 8.3% loss in the individual therapy group. By contrast, Ball and Mitchell (2004) did not detect differences in weight change; however, the small sample size and broader age range (*M*_age_ = 18.9 years; range 13–23 years) limits interpretability of these null findings. Psychological symptom change was generally similar across arms, and where reported, effect sizes were small with wide confidence intervals, suggesting either minimal differences or insufficient power.

### 3.2 Research Question 2: Outpatient Eating Disorder‐Focused Single‐Family Therapies Versus Other Family Therapies

To address the second research question, five RCTs were identified that compared eating disorder‐focused single‐family therapies (such as FBT or FT‐AN) with other family‐based approaches. These alternative models included MFT (MFT‐AN), SyFT, and formats where parents and adolescents are seen separately (Separated Family Therapy [SFT] or Parent‐Focused Treatment [PFT]). Table 3 displays the study characteristics of the included studies.

**TABLE 3:** Outpatient eating disorder‐focused single‐family therapies versus other family therapies.

Study	Demographics	Family therapy	Compared to	*n* (intervention)	*n* (comparison)
Agras et al. (2014)	Mean age = 15.3 years (SD = 1.8) Age range = 12–18 years Female = 89.2% Race/ethnicity = White 79.1%, Hispanic 10.1%, Asian 5.1%, mix 5.7% SES = NR	FBT	Systemic family therapy (SyFT)	79	80
Eisler et al. (2000, 2007)	Mean age = 15.6 years (SD = 1.3) Age range = 12–18 years Female = 97.5% Race/ethnicity = NR SES = social class (Hollingshead classification system) I–II 65%, III–V 22.5%, VI–VIII	FT‐AN/conjoint family therapy^a^	Separated family therapy	19	21
Eisler et al. (2016)	Mean age = 15.7 years (SD = 1.7) Age range = 13–20 years Female = 91% Race/ethnicity = White 90%, other 3.5% Education = pre‐GCSE 70%, post‐GCSE 28.7% Employment = NR Income = NR	FT‐AN/conjoint family therapy^a^	MFT‐AN	82	85
Le Grange et al. (1992)	Mean age = 15.33 years (SD = 1.81) Age range = 12–17 years Female = 88.88% Race/ethnicity = NR SES = NR	Conjoint family therapy^b^	Family counseling	9	9
Le Grange et al. (2016)	Mean age = 15.5 years (SD = 1.5) Age range = 12–18 years Female = 87.7% Race/ethnicity = NR SES = parental university degree: mother 37.8%, father 38.2%	FBT	Parent‐focused treatment	55	51

^a^ Eisler et al. (2016) used several terms (FT‐AN/conjoint family therapy) to describe a manualized single‐family therapy.

^b^ The family therapy in Le Grange et al. (1992) is a precursor of manualized FBT.

Across the five randomized trials comparing outpatient single‐family therapies (e.g., FBT, FT‐AN, BFST) with other family‐based approaches for adolescents with AN, considerable variability was observed in study design, treatment format, and methodological quality (see Table 3). Sample sizes ranged widely from very small pilot studies (e.g., Le Grange et al. 1992; *n* = 9 per arm) to larger, adequately powered multi‐site trials (e.g., Agras et al. 2014; *N* = 159; Eisler et al. 2016; *N* = 167). Mean participant age was generally mid‐adolescence (≈15–16 years), though age ranges sometimes extended into early adulthood (up to 20 years), which may limit comparability across trials.

The interventions also differed in format: some evaluated manualized eating‐disorder‐focused single‐family treatments, such as FBT or FT‐AN, whereas the comparator arms included more MFT (MFT‐AN), SyFT, PFT, or SFT. Outcome domains also varied considerably (Table 4): while all studies assessed medical or somatic outcomes (e.g., BMI, % ideal body weight, Morgan–Russell nutritional status), only some included remission rates, rehospitalization, or a broad range of psychological outcomes such as EDE‐Q subscales, depressive symptoms (BDI/CDI), self‐esteem (RSES), or quality‐of‐life measures. Follow‐up periods ranged from post‐treatment to 12‐month follow‐up, and reporting of attrition and adherence was inconsistent across studies.

**TABLE 4:** Detailed overview of outcome measures: outpatient eating disorder‐focused single‐family therapies versus other family therapies.

Study	Type of outcome (medical/somatic, psychological, remission, rehospitalization)	Outcome measure/instrument	Hedges *g* ^a^ [95% CI] posttest (PT) or follow‐up (F‐U)
Agras et al. (2014)	Remission	Remission rate	0.23, 95% CI [−0.08, 0.55] (PT) 0.05, 95% CI [−0.26, 0.36] (F‐U)
	Medical/somatic	% Ideal body weight	−0.00, 95% CI [−0.32, 0.31] (PT) 0.01, 95% CI [−0.31, 0.32] (F‐U)
	Psychological	EDE‐Q (composite)	−0.03, 95% CI [−0.35, 0.28] (PT) −0.01, 95% CI [−0.33, 0.30] (F‐U)
		BDI	−0.23, 95% CI [−0.54, 0.08] (PT) −0.25, 95% CI [−0.57, 0.06] (F‐U)
		RSES	0.43, 95% CI [0.11, 0.74] (PT) 0.50, 95% CI [0.18, 0.81] (F‐U)
		STAI	−0.19, 95% CI [−0.51, 0.12] (PT) −0.22, 95% CI [−0.54, 0.09] (F‐U)
		QLES	−0.16, 95% CI [−0.47, 0.15] (PT) −0.19, 95% CI [−0.50, 0.12] (F‐U)
		Child Yale‐Brown Obsessive Compulsive Scale	0.02, 95% CI [−0.30, 0.33] (PT) 0.04, 95% CI [−0.28, 0.35] (F‐U)
		Yale‐Brown‐Cornell Eating Disorder Scale	−0.01, 95% CI [−0.32, 0.30] (PT) 0.01, 95% CI [−0.30, 0.32] (F‐U)
Eisler et al. (2000, 2007)	Medical/somatic	Weight (%abw)	−0.42, 95% CI [−1.05, 0.21] (PT) −0.56, 95% CI [−1.21, 0.09] (F‐U)
		BMI	−0.48, 95% CI [−1.11, 0.15] (PT)
		Morgan Russell Assessment Schedule—Nutritional status	−0.20, 95% CI [−0.82, 0.43] (PT)
		Morgan Russell Assessment Schedule—Menstrual function	−0.19, 95% CI [−0.81, 0.43] (PT)
	Psychological	Morgan Russell Assessment Schedule—binary outcome (composite)^b^	−0.76, 95% CI [−1.41, −0.12] (PT) −0.51, 95% CI [−1.15, 0.14] (F‐U)
		Morgan Russell Assessment Schedule—Mental State	0.66, 95% CI [0.03, 1.30] (PT)
		Morgan Russell Assessment Schedule—Psychosexual adjustment	0.45, 95% CI [−0.18, 1.07] (PT)
		EAT	−0.10, 95% CI [−0.72, 0.52] (PT)
		EDI (composite)	0.39, 95% CI [−0.24, 1.01] (PT)
		RSES	0.56, 95% CI [−0.07, 1.20] (PT)
		Depression	0.27, 95% CI [−0.36, 0.89] (PT)
Eisler et al. (2016)	Medical/somatic	% Mean BMI	−0.25, 95% CI [−0.55, 0.05] (PT) −0.40, 95% CI [−0.70, −0.09] (F‐U)
	Psychological	Morgan Russell Assessment Schedule—binary outcome (composite)^b^	−0.36, 95% CI [−0.67, −0.06] (PT) −0.26, 95% CI [−0.57, 0.04] (F‐U)
		EDE‐Q Restraint	0.20, 95% CI [−0.10, 0.51] (PT) 0.19, 95% CI [−0.11, 0.50] (F‐U)
		EDE‐Q Eating Concern	0.07, 95% CI [−0.24, 0.37] (PT) 0.09, 95% CI [−0.21, 0.39] (F‐U)
		EDE‐Q Shape Concern	0.23, 95% CI [−0.08, 0.53] (PT) 0.22, 95% CI [−0.08, 0.53] (F‐U)
		EDE‐Q Weight Concern	0.19, 95% CI [−0.12, 0.49] (PT) 0.19, 95% CI [−0.12, 0.49] (F‐U)
		BDI	0.15, 95% CI [−0.16, 0.45] (PT) 0.10, 95% CI [−0.20, 0.41] (F‐U)
		RSES	0.12, 95% CI [−0.19, 0.42] (PT) −0.05, 95% CI [−0.35, 0.25] (F‐U)
Le Grange et al. (1992)	Medical/somatic	Weight (% abw)	0.33, 95% CI [−0.60, 1.26] (F‐U)
	Psychological	Morgan Russell Assessment Schedule (composite)^b^	0.29, 95% CI [−0.64, 1.22] (F‐U)
		EAT	0.03, 95% CI [−0.90, 0.95] (F‐U)
		RSES	0.00, 95% CI [−0.92, 0.93] (F‐U)
Le Grange et al. (2016)	Remission	Remission rate	−0.60, 95% CI [−0.99, −0.21] (PT) −0.50, 95% CI [−0.89, −0.11] (F‐U 6 m) −0.22, 95% CI [−0.60, 0.16] (F‐U 12 m)
	Rehospitalization	Number of hospitalizations	−0.46, 95% CI [−0.85, −0.08] (PT) −0.40, 95% CI [−0.91, 0.11] (FU 6 m)
	Medical/somatic	% Mean BMI	−0.03, 95% CI [−0.41, 0.35] (PT) 0.10, 95% CI [−0.28, 0.48] (F‐U 6 m) 0.07, 95% CI [−0.31, 0.45] (F‐U 12 m)
	Psychological	EDE‐Q (global score)	−0.16, 95% CI [−0.54, 0.22] (PT) −0.14, 95% CI [−0.52, 0.24] (F‐U 6 m) −0.13, 95% CI [−0.51, 0.25] (F‐U 12 m)
		EDE‐Q Restraint (subscale)	−0.21, 95% CI [−0.59, 0.17] (PT) −0.27, 95% CI [−0.65, 0.11] (F‐U 6 m) −0.20, 95% CI [−0.58, 0.18] (F‐U 12 m)
		EDE‐Q Eating Concerns (subscale)	−0.13, 95% CI [−0.51, 0.26] (PT) −0.05, 95% CI [−0.43, 0.33] (F‐U 6 m) −0.12, 95% CI [−0.50, 0.26] (F‐U 12 m)
		EDE‐Q Weight Concerns (subscale)	−0.26, 95% CI [−0.64, 0.12] (PT) −0.29, 95% CI [−0.67, 0.10] (F‐U 6 m) −0.13, 95% CI [−0.51, 0.25] (F‐U 12 m)
		EDE‐Q Shape Concerns (subscale)	−0.25, 95% CI [−0.63, 0.13] (PT) −0.25, 95% CI [−0.63, 0.14] (F‐U 6 m) −0.20, 95% CI [−0.58, 0.18] (F‐U 12 m)
		CDI	−0.11, 95% CI [−0.49, 0.27] (PT) 0.01, 95% CI [−0.37, 0.39] (F‐U 6 m) −0.42, 95% CI [−0.81, −0.04] (F‐U 6 m)
		RSES	−0.16, 95% CI [−0.54, 0.22] (PT) 0.03, 95% CI [−0.35, 0.41] (F‐U 6 m) −0.23, 95% CI [−0.61, 0.15] (F‐U 12 m)

Abbreviations: BDI, Beck Depression Inventory; CDI, Child Depression Inventory; EAT, Eating Attitudes Test; EDE‐Q, Eating Disorders Examination; EDI‐2, Eating Disorders Inventory; F‐U, follow‐up; PT, post‐test; QLES, Quality of Life and Enjoyment Scale; RSEF, Rosenberg Self Esteem Scale; STAI, State–Trait Anxiety Inventory.

^a^ Hedges' *g*, 0.20 = small, 0.50 = moderate, 0.80 = large, respectively (Cohen 1988).

^b^ The Morgan–Russell Assessment Schedule composite includes both medical and psychological subscales.

In terms of methodological quality, the larger recent trials (Agras et al. 2014; Eisler et al. 2016; Le Grange et al. 2016) generally provided adequate power to detect at least moderate effects and applied clearer diagnostic and remission criteria, whereas earlier studies often suffered from small sample sizes, limited blinding of outcome assessors, and incomplete reporting of allocation and attrition.

Regarding the effects of family interventions on AN recovery, all treatment formats demonstrated substantial effectiveness in promoting weight restoration and reducing eating disorder symptoms. For instance, in the largest study comparing single‐family therapy to MFT format (Eisler et al. 2016; *N* = 169), both FT‐AN and MFT‐AN led to clinically significant improvements. Similarly, Agras et al. (2014) found no significant differences in remission rates at the end of treatment or at follow‐up between FBT and SyFT, a therapy that does not directly focus on eating behaviors. However, certain studies indicate that alternative formats may offer specific advantages in particular contexts. Eisler et al. (2016) reported a statistically significant short‐term advantage for MFT‐AN, with a higher proportion of patients achieving a good or intermediate outcome at the end of treatment (75% vs. 60%). This initial benefit, which was not maintained at the 18‐month follow‐up, might be attributed to the peer support and shared learning inherent in the multi‐family format.

### 3.3 Research Question 3: Outpatient Individual Psychological Treatment

In line with treatment guidelines that position individual therapy as a second‐line or adjunctive approach, the evidence base for standalone individual psychological treatments for adolescents with AN is sparse. Beyond the five RCTs that used individual therapy as a comparator for family therapy (as discussed under RQ1), two additional RCTs evaluating individual psychological interventions were identified (Table 5).

**TABLE 5:** Studies evaluating individual outpatient psychological interventions for adolescent AN.

Study	Demographics	Individual therapy	Compared to	*n* (intervention)	*n* (comparison)
Balzan et al. (2023)	Mean age = 15.2 years (SD = 1.3) Age range = 12–18 years Female = 89.2% Race/ethnicity = White (79.1%), Hispanic 10.1%, Asian 5.1%, mix 5.7% SES = NR	Metacognitive training (MCT)	TAU (= CBT/FBT)	17	12
Lock et al. (2018)	Mean age = 14.49 years (SD = 1.64) Age range = 12–18 years Female = 90% Race/ethnicity = Caucasian 60%, Asian 16.7%, more than one race 23.3%; Hispanic 30% SES = NR	Art Therapy (AT) + FBT	CRT + FBT	15	15

The two identified studies were small‐scale, underpowered feasibility trials that evaluated adjunctive individual therapies delivered in combination with FBT, rather than as standalone interventions. Both studies examined the added value of targeted individual modules within FBT, aiming to address specific patient subgroups or treatment needs. Lock et al. (2018; *N* = 30) conducted a feasibility trial for adolescents with AN and high co‐occurring obsessive‐compulsive (OC) traits, a group known to respond less favorably to FBT alone. This study compared the addition of either Art Therapy (AT) or Cognitive Remediation Therapy (CRT) to a standard FBT protocol. While the study was not powered to detect statistically significant differences, both combined treatments demonstrated notable reductions in OC traits and facilitated weight gain. The findings suggest that for patients with significant OC features, augmenting FBT with an individual therapy targeting cognitive or emotional expression is a feasible and potentially beneficial approach.

Similarly, Balzan et al. (2023; *N* = 35) investigated the addition of MCT to TAU (which included FBT or CBT for some participants). This pilot study found that the addition of MCT led to a statistically significant short‐term reduction in “concern over mistakes,” a core component of perfectionism. However, this effect was not maintained at the 3‐month follow‐up, and no significant differences were found for broader eating disorder pathology.

### 3.4 Research Question 4: Inpatient Treatment Versus Outpatient or Day Patient Treatment

Three RCTs were identified that compared the effectiveness of a complete inpatient treatment program with less intensive settings, such as specialist outpatient care, day‐patient programs, or standard community‐based services (Table 6).

**TABLE 6:** Inpatient treatment programs (complete program).

Study	Demographics	Intervention	Comparison condition	*n* (intervention)	*n* (comparison)
Gowers et al. (2007)	Mean age = 14.9 years (SD = unknown) Age range = 12–18 years Female = 95% Race/ethnicity = NR SES = NR	Two outpatient arms combined: 1. General CAMHS (*n* = 55); 2. Specialist out‐patient (*n* = 55)	Inpatient treatment (*n* = 57)	110	57
Herpertz‐Dahlmann et al. (2014)	Mean age inpatient/day patient = 15.2 years (SD = unknown)/15.3 (SD = 1.5) Age range = 11–18 years Female = 100% Race/ethnicity = NR SES inpatient/day Patient = MRAOS scale E (socioeconomic status) 8.7 (2.7)/9.3 (2.1)	3 weeks inpatient + day patient treatment	3 weeks inpatient + continued inpatient treatment	86	75
Madden et al. (2015)	Mean age = 14.9 years (SD = 1.46) Age range = 12–18 years Female = 95% Race/ethnicity = White 82.9%, Asian 12.2%, other 4.9% SES = NR	Short hospitalization (medical stability) + outpatient FBT	Long hospitalization (weight recovery) + outpatient FBT	41	41

The largest of the RCTs, a multi‐center trial by Gowers et al. (2007; *N* = 167), compared a 6‐week multimodal inpatient program against both specialist outpatient treatment and TAU in community clinics. At both 1‐ and 2‐year follow‐ups, no significant differences in outcomes were found between the three groups. Critically, the study noted that adolescents who were randomized to but refused inpatient treatment, and thus continued with outpatient care, had significantly better outcomes at 1‐year follow‐up than those who were admitted. This finding challenges the assumption that inpatient care is inherently superior, suggesting that patient motivation and acceptance of the treatment setting are crucial factors. However, interpretation is limited by high crossover rates, variable treatment adherence (≈49% inpatient vs. 70%–75% outpatient), and differences in treatment intensity between groups. These factors likely reduced detectable differences in the intention‐to‐treat analyses and make it difficult to draw firm conclusions about the true effects of each treatment.

Two non‐inferiority trials demonstrated that a brief initial inpatient admission followed by a step‐down to a less intensive setting produced outcomes equivalent to continuous, prolonged hospitalization. Herpertz‐Dahlmann et al. (2014) conducted a large multicenter randomized non‐inferiority trial (*N* = 172) using standardized treatment protocols across sites, strengthening internal validity. The researchers found that a brief 3‐week inpatient stay followed by day‐patient treatment was as effective for weight gain and maintenance as continuous inpatient care, while also being 20% more cost‐effective. Similarly, Madden et al. (2015) compared a short hospitalization focused only on medical stabilization against a longer admission for full weight restoration, with both groups receiving FBT post‐discharge. The study of Madden et al. (2015) was an RCT with blinded outcome assessment, standardized inpatient protocols, and manualized FBT with fidelity checks, strengths that reduce measurement bias. The study found no significant differences in remission rates or subsequent hospital days, but the shorter‐stay group had significantly fewer total hospital days over the 12‐month period, indicating greater cost‐effectiveness. A moderator analysis also suggested that adolescents with comorbid OCD had better outcomes with the shorter hospital stay.

### 3.5 Research Question 5: Psychological Treatment Modules Within Inpatient Programs

Seven RCTs were identified that evaluated the effectiveness of specific psychological group modules added to standard inpatient TAU (Table 7). These adjunctive therapies aimed to target core underlying mechanisms of AN, such as body image distress, low self‐esteem, cognitive inflexibility, and compulsive exercise.

**TABLE 7:** Psychological treatment modules implemented during inpatient programs for AN.

Study	Demographics	Inpatient module	Control condition	*n* (intervention)	*n* (comparison)
Biney et al. (2021)	Mean age = 14.2 years (SD = 1.6) Age range = 11–17 years Female = 100% Race/ethnicity = NR SES = NR	Practical body image therapy (PBI)	TAU	15	16
Biney et al. (2022)	Mean age = 15.22 years (SD = 1.62) Age range = 12–17 years Female = 100% Race/ethnicity = NR SES = NR	Self‐esteem group therapy	TAU	15	14
Crevits et al. (2024)	Mean age CBT‐I/TAU = 15.51 years (SD = 1.2)/15.65 (1.52) Age Range = “adolescents” Female = NR Race/ethnicity = NR SES = NR	CBT for insomnia (CBT‐I)	TAU	22	11
Dikstein et al. (2023)	Mean age Completers = 17.9 years (SD = 3.61) Age range = 12–25 years Female = 100% Race/ethnicity = NR SES = NR	Attention modification treatment (ABMT)	TAU	39 (ED)/38 (ANX)	33
Dittmer et al. (2020)	Mean age HEB/TAU = 20.04 years (SD = 5.7)/18.32 (5.19) Age range = 13–45 years Female = 100% Race/ethnicity = NR SES = NR	Healthy exercise behavior treatment (HEB)	TAU	112	95
Giombini et al. (2021)	Mean age = 14.49 years (SD = 1.75) Age range = 10–18 years Female = 93.8% Race/ethnicity = White British or other White 91.4%, Asian 7.5%, mixed White and Black Caribbean 1.3% SES = NR	CRT immediate + TAU		40^a^	
		CRT delayed + TAU		40^a^	
Herbrich‐Bowe et al. (2022)	Mean age = 15.1 years (SD = 1.5) Age range = 11–17 years Female = 100% Race/ethnicity = NR SES = low 2%, middle 64%, high 27%	CRT	TAU + non‐specific cognitive training (NSCT)	28	28

Abbreviation: CRT, Cognitive Remediation Therapy.

^a^ This study had a delayed‐start design; the groups were divided in two and both received the treatment at a different onset of time.

The current evidence on adjunctive psychological modules delivered during inpatient care for adolescents with AN (Table 8) is characterized by significant methodological limitations. Most available studies are small‐scale pilot trials often without preregistered protocols, with sample sizes typically ranging from 20 to 40 per arm, with the exception of the study of Dittmer (total *N* > 200). Across studies, treatment fidelity and blinding procedures were rarely reported, and dropout rates frequently exceeded 30%–40%, raising concerns about internal validity and selective attrition. Several interventions were tested within complex, multi‐component inpatient programs, making it difficult to isolate the specific effects of the added module from standard therapeutic activities already in place. This confounding factor was noted by several authors (e.g., Biney et al. 2021; Dikstein et al. 2023) as a primary reason for the lack of significant findings in their studies. Moreover, follow‐up assessments were scarce or absent, leaving uncertainty about the durability of observed gains. Together, these factors indicate that, although the reviewed studies offer valuable preliminary insights, the overall quality of the evidence remains low, and findings should be interpreted with caution.

**TABLE 8:** Overview of psychological treatment modules implemented during inpatient care for adolescents with AN.

Intervention	Description
Practical body image therapy	The focus of this intervention is to reduce body image distress and avoidance in adolescents with AN. This 10‐week manualized group intervention combines psychoeducation, cognitive restructuring, and experiential exercises such as guided mirror exposure and behavioral tasks related to clothing and appearance. It aims to foster a more accepting and flexible body image.
Self‐esteem group therapy	The focus of this intervention is to strengthen self‐esteem in adolescents with AN. This six‐session manualized group program uses cognitive‐behavioral strategies to challenge negative self‐beliefs and promote self‐acceptance. It includes exercises targeting self‐evaluation, positive self‐attributes, and social comparison.
CBT for insomnia	The focus of this intervention is to improve sleep quality and reduce insomnia‐related impairments in adolescents with AN. This structured cognitive‐behavioral intervention addresses maladaptive sleep habits through psychoeducation, sleep hygiene training, stimulus control, and cognitive restructuring. It is designed to restore healthy sleep patterns and improve physical well‐being.
Attention bias modification treatment	This intervention focuses on reducing attentional bias toward threat‐related stimuli (e.g., body or anxiety cues) in adolescents with AN. This computer‐based intervention uses dot‐probe tasks to retrain attention away from disorder‐relevant stimuli. The goal is to reduce anxiety and stress reactivity that may maintain disordered eating behaviors.
Healthy exercise behavior	The focus of this intervention is to reduce compulsive exercise behaviors and promote a healthier relationship with physical activity. This group‐based intervention includes psychoeducation about exercise and energy balance, self‐monitoring, and behavioral experiments to support flexible and balanced exercise habits.
Cognitive remediation therapy	The focus of CRT is to improve neurocognitive functioning in adolescents with AN, particularly cognitive flexibility and central coherence. This individually delivered intervention uses tasks such as pattern recognition and problem‐solving combined with reflection to increase metacognitive awareness.

Regarding the available trials, two small‐scale studies by Biney et al. (Biney et al. 2021) evaluated inpatient modules targeting body image and self‐esteem, respectively. Both reported small‐to‐moderate improvements on their primary outcomes compared with the control condition, although these effects were not consistently statistically significant or sustained at follow‐up. Similarly, a recent trial of CBT for insomnia (CBT‐I) in adolescents with AN (Crevits et al. 2024) reported significant improvements in sleep onset latency and overall sleep quality, yet these benefits did not extend to eating‐disorder symptoms or psychological well‐being. In the largest study of this type, Dittmer et al. (2020) found that a specialized intervention for “healthy exercise behavior” significantly reduced compulsive exercise, but this did not translate into improvements in weight gain or eating disorder cognitions compared to the control group.

Three separate RCTs investigated the added value of Cognitive Remediation Therapy (CRT), an intervention designed to improve cognitive flexibility and central coherence (Giombini et al. 2021; Herbrich‐Bowe et al. 2022; Dikstein et al. 2023). None of these studies found CRT to be superior to a control condition (either TAU or a non‐specific cognitive training). For example, Herbrich‐Bowe et al. (2022) found no significant differences in cognitive flexibility or central coherence and noted that the non‐specific training was actually superior in improving self‐reported planning abilities. Overall, the inpatient modules appear to target specific components of functioning effectively, such as sleep, exercise behavior, or cognitive style, but there is no evidence that these effects translate into broader improvements in core eating disorder symptoms or overall psychological recovery.

## 4 Discussion

This study aimed to provide a critical review of the evidence for psychological treatments for adolescents with AN across various care settings, consistent with international treatment guidelines. We formulated five research questions which we further discuss in detail below. In summary, the findings reveal significant gaps in the existing research and highlight the considerable challenges involved in conducting robust studies on psychological treatments for adolescents with AN, a population that remains critically underserved despite the severe consequences of the disorder. These results underscore an urgent need for methodologically rigorous research to enhance the scientific foundation, advance clinical practice, and refine international treatment guidelines to better address the unique and complex needs of adolescents with AN.

### 4.1 Research Question 1: Outpatient Family Therapy Versus Individual Therapy

The first research question examined the effects of eating‐disorder‐focused family therapies, considered the first‐line treatment for adolescents with AN, relative to individual therapy approaches. Only four RCTs met the inclusion criteria. Hence, the evidence rests on only four, largely underpowered, RCTs. We consider this a small body of research in this population, given that AN typically emerges during adolescence and is associated with severe morbidity, chronic impairment, and substantial impact on quality of life. Moreover, treatment selection in this population entails a fundamental distinction between individual and family‐based approaches, which differ in how responsibility and motivation for change are shared between the adolescent and the family. This underscores the need for stronger evidence to guide treatment selection. Overall, the evidence points to a modest but clinically relevant advantage of family over individual therapy for weight restoration and reduced need for hospitalization, while psychological outcomes appear largely equivalent.

Beyond the small number of trials, several methodological limitations constrain the strength of the evidence. Three of the four studies were underpowered, with sample sizes of 18, 21, 37, and 124 participants, limiting their ability to detect small‐to‐moderate effects. All trials focused exclusively on single‐family models (FBT or BFST). Study designs were heterogeneous: some trials evaluated outpatient treatment from the start of care, others examined family therapy as a maintenance intervention after inpatient weight restoration, and the individual‐therapy comparators ranged from supportive psychotherapy to ego‐oriented or CBT‐like approaches. In addition, the outcome domains and follow‐up periods varied considerably across studies, encompassing diverse weight‐related indices, remission criteria, hospitalization rates, and psychological measures, with follow‐up ranging from end‐of‐treatment to 12 months. This heterogeneity, together with small samples, makes it difficult to synthesize findings and to establish consistent evidence for either medical or psychological outcomes.

Several key questions remain before robust, evidence‐based treatment recommendations can be made. Notably, no sufficiently powered RCTs have directly compared the effectiveness of family therapy with individual therapy protocols that are common in adults such as CBT‐E or MANTRA. Although these therapies have been applied successfully in adolescent populations (e.g., Le Grange et al. 2020; Wittek et al. 2023), evidence from head‐to‐head trials comparing them with family therapy is lacking, leaving an important gap in understanding how these approaches differ in effectiveness for younger patients.

Additionally, there is limited research on factors contributing to treatment success or on indicators guiding clinicians in choosing between caregiver‐led interventions (e.g., FBT) and approaches emphasizing individual ownership (e.g., CBT‐E or AFT). Recent findings highlight the importance of tailoring treatment based on family and patient characteristics. An RCT by Lock et al. (2024) showed that baseline parental self‐efficacy predicts early treatment response, and that an adaptive strategy, Intensive Parental Coaching, improved outcomes for families with initially low self‐efficacy. This underscores the relevance of parent‐specific factors in selecting interventions. A study by Le Grange et al. (2020), using a shared decision‐making approach, found that lower‐weight patients who chose CBT‐E over FBT were generally older, had more severe depression, longer illness duration, greater impairment, and more prior treatment. While FBT facilitated early weight gain, both CBT‐E and FBT showed similar outcomes in ED psychopathology and secondary measures at follow‐up. These findings call for further research into how patient profiles influence treatment effectiveness.

### 4.2 Research Question 2: Evaluating Types of Family Therapy

The second question concerned the effectiveness of outpatient single‐family therapies, such as FBT and BFST, for adolescents with AN compared to other family‐based approaches, including MFT and SyFT. Across five RCTs, nuanced insights emerged regarding the relative effectiveness of these formats. Overall, both single‐family therapies (e.g., FBT, FT‐AN) and alternative models (e.g., MFT, SyFT, PFT) demonstrated substantial effectiveness in promoting weight restoration and reducing eating disorder symptoms. Taken together with the RCTs included in Research Question 1, family‐based interventions for adolescent AN show a consistent pattern of small‐ to large‐sized positive effects on weight restoration, remission rates, hospitalization rates, and core eating‐disorder symptoms, regardless of the control condition. These findings highlight the overall impact and importance of family‐based interventions in the treatment of adolescents with AN.

However, certain studies suggest that alternative formats may offer specific advantages in particular clinical contexts. For instance, MFT‐AN was associated with greater short‐term gains than FT‐AN, potentially due to its emphasis on peer support, shared learning, and family engagement. Similarly, SFT and PFT appeared especially beneficial for families with high levels of parental criticism or caregiver distress – contexts in which separating parent and adolescent sessions may reduce conflict and enhance responsiveness. The persistence of improved outcomes in SFT for high‐criticism families, along with trends toward higher (i.e., faster) remission in PFT compared to FBT, highlights the relevance of tailoring treatment to family dynamics. These findings also point to the moderating role of family environment and parental behavior in treatment outcomes. While no single format emerged as consistently superior across all studies or follow‐up periods, the evidence underscores the value of offering a range of family‐based options and aligning treatment selection with both clinical severity and family‐specific characteristics.

That said, the practical implications are more complex. The training and expertise required to implement multiple specialized treatments present significant resource challenges, particularly given the limited and inconsistent evidence that any specific model confers substantial differential benefit. Rather than proliferating formats, the core issue may lie in our limited understanding of what works for whom: a gap that future research must address to meaningfully inform personalized care.

### 4.3 Research Question 3: Individual Psychological Interventions for Adolescent AN

Research Question 3 examined the effectiveness of outpatient individual psychological therapies on recovery and long‐term outcomes for adolescents with AN. Evidence from this review shows that RCTs in this area are scarce. Beyond the five trials comparing family‐based therapy (FBT or BFST) with individual therapy, only two additional feasibility RCTs were identified. Zooming in on the findings, both RCTs evaluated additive individual interventions delivered alongside family‐based approaches rather than as standalone therapies. Lock et al. (2018) tested the addition of CRT or Art Therapy to FBT in adolescents with obsessive‐compulsive traits, finding both approaches feasible and acceptable, with some improvement in obsessive‐compulsive symptoms. While the addition of such modules appeared to improve cognitive flexibility and maladaptive thinking patterns, these cognitive gains did not translate into significant improvements on core eating‐disorder outcomes. Balzan et al. (2023) evaluated an online MCT program (MCT‐ED) added to treatment as usual, showing good retention and short‐term reductions in perfectionism, but no sustained or broader effects. Together, these studies provide early support for the feasibility and acceptability of integrating targeted cognitive modules within family‐based frameworks, though their small samples and pilot designs limit conclusions about efficacy.

### 4.4 Research Question 4: Complete Inpatient Treatment Programs

Only three studies have evaluated inpatient treatment programs for adolescents with AN, reflecting a severely limited evidence base. Conducting RCTs in this setting poses substantial ethical and methodological challenges, as current treatment guidelines emphasize that inpatient care should be reserved for cases of absolute necessity, for example, when outpatient treatment has failed or when other complex clinical or contextual factors warrant hospitalization (Gowers et al. 2007; Madden et al. 2015). Randomly assigning patients to inpatient versus outpatient treatment is therefore often neither feasible nor ethically appropriate, given the high level of clinical risk in this population. These challenges were evident in one of the RCTs (Gowers et al. 2007), in which high rates of non‐adherence and crossover between treatment arms substantially limited the interpretability of the findings. Given both the paucity of evidence and the known adverse effects of inpatient programs, such as social isolation, perceived coercion, disruption of daily life, and potential reinforcement of illness behaviors, decisions regarding hospitalization must carefully weigh patient motivation, family resources, and the potential psychosocial costs of admission (Gowers et al. 2007).

Evidence so far suggests that shorter inpatient stays focused on medical stabilization, followed by day treatment or outpatient care, may be more cost‐effective, safe, and effective than prolonged hospitalization (Herpertz‐Dahlmann et al. 2014; Madden et al. 2015). Longer inpatient stays did not result in significant improvements in outcomes such as weight restoration or reduced relapse rates. For example, one study concluded that day patient treatment could serve as a viable, less costly alternative to extended inpatient care, offering comparable outcomes in terms of safety and effectiveness (Herpertz‐Dahlmann et al. 2014). These findings support a shift toward shorter, more targeted inpatient interventions complemented by robust outpatient support.

### 4.5 Research Question 5: Inpatient Psychological Treatment Programs

Several psychological interventions have been tested in inpatient settings for adolescents with AN, though evidence remains limited and mixed. A structured “healthy exercise” program significantly reduced compulsive exercise behaviors in a large sample but did not affect weight restoration, ED cognitions, or general psychopathology. Two studies evaluating CRT yielded similarly inconclusive results: in both, NSCT produced equal or greater improvements in executive functioning, such as planning and organization (Giombini et al. 2021; Herbrich‐Bowe et al. 2022). One CRT study aimed to enhance central coherence but found no significant effects overall, though outcomes appeared moderated by autistic traits.

While the addition of targeted psychological modules to inpatient care is conceptually promising, the available evidence is preliminary and methodologically weak. Reported improvements are confined to narrow symptom domains and have not translated into broader recovery. The small sample sizes, inconsistent control conditions, and absence of long‐term follow‐up across studies preclude firm conclusions about efficacy (Gowers et al. 2007; Herpertz‐Dahlmann et al. 2014; Madden et al. 2015).

Inpatient treatment remains essential for medical stabilization but is often perceived by adolescents and parents as coercive, which can undermine engagement and motivation. The disruption of social connections with peers and family during admission may also negatively impact patients' social competence and emotional well‐being (Guarda et al. 2007; Nyttingnes et al. 2018). Beyond physical recovery, greater emphasis on psychological and relational processes may enhance outcomes and foster patient agency and long‐term resilience (Lock and Le Grange 2019; Treasure et al. 2020). Strikingly, none of the identified RCTs evaluated interventions or modules aimed at strengthening parent involvement or improving parent–child interactions during inpatient care. Given that adolescents typically return to their family environment after discharge, integrating family‐based principles into inpatient programs appears a critical next step to support continuity of care and reduce relapse risk.

### 4.6 Strengths and Limitations

To our knowledge, no prior study has systematically provided a complete overview of the published RCTs on the effectiveness of psychological interventions for youth with AN while focusing on treatment recommendations outlined in international guidelines. The current review allowed us to identify and highlight existing research gaps in the field. However, several limitations should be considered. Our conclusions are constrained by significant methodological limitations inherent in the primary studies that were included in the syntheses. The pooled effect size estimate and moderator analyses that address Question 1 are based on just five RCTs, of which four were statistically underpowered, limiting the generalizability of findings. The small sample sizes and heterogeneous designs precluded more robust moderator analyses, further restricting the interpretation of outcomes.

It is important to note that a few published RCTs were not included, as these did not align with the objectives of our research questions (Nyman‐Carlsson et al. 2020; Onnis et al. 2012). Also, attrition in primary studies is a considerable challenge, impacting the quality of long‐term evaluations of treatment effectiveness. This highlights the difficulty of retaining participants, particularly in outpatient settings, and underscores the need for strategies like multiple imputation and intensive follow‐up efforts to mitigate this issue. Attrition can significantly bias research findings, especially if those lost at follow‐up systematically differ from those who remain.

Variation in diagnostic criteria across studies (e.g., differing use of DSM or ICD systems, or clinician‐defined diagnoses) may have contributed to diagnostic heterogeneity, further complicating comparisons. Additionally, the heterogeneity of outcome measures and control conditions used across primary studies further limits the generalizability and comparability of findings. Many studies focused primarily on weight restoration as a primary outcome, neglecting other crucial aspects like psychological well‐being, quality of life, and long‐term functioning. While the evidence supports the effectiveness of FBT in achieving early weight restoration compared to individual therapies, the limited scope and underpowered nature of the available RCTs highlight the need for larger, methodologically rigorous trials. These trials should aim to elucidate not only the somatic but also the long‐term psychological and functional benefits of FBT, offering a more comprehensive understanding of its role in treating adolescent AN.

### 4.7 Future Research

Despite substantial ongoing efforts, this study revealed significant gaps in our understanding of effective treatments for AN during adolescence, the phase in which AN most commonly peaks. To lay a stronger foundation for future treatment guidelines for youth with AN, several key recommendations should be considered. First, future research should prioritize consistency in study design and control conditions. This includes the adoption of standardized intervention protocols, clear definition and detailed reporting of treatment components, and the use of consistent somatic and psychological outcome measures across studies. Additionally, our review revealed that the vast majority of existing RCTs are underpowered, limiting the reliability and interpretability of their findings. This partly reflects that most RCTs were pilot studies, deliberately underpowered and designed primarily to assess feasibility rather than efficacy. However, the resulting lack of statistical power substantially limits confidence in the reported effect sizes and contributes to inconsistencies across studies, preventing the development of clear clinical guidance. Without adequately powered trials, the field continues to rely on fragmented and inconclusive evidence, making it difficult to determine the relative effectiveness of different interventions.

Second, current evidence highlights the importance of identifying factors that influence which adolescents respond best to particular treatment approaches. Existing RCTs suggest that treatment outcomes may differ across patient and family subgroups, for example, among adolescents with prominent obsessive‐compulsive traits (Le Grange et al. 2016) or families characterized by high maternal criticism (Eisler et al. 2000). Future studies should therefore prioritize clarifying such moderators of treatment response, including age, illness duration, comorbidities, and family dynamics. Advancing this knowledge would allow for more informed treatment matching and incremental adaptation of existing family‐ and individual‐based therapies, with the ultimate aim of improving long‐term outcomes and reducing relapse risk.

Moving beyond a one‐size‐fits‐all approach may involve combining elements of different therapies and integrated approaches, such as integrating cognitive techniques into FBT or adapting interventions to address specific challenges, such as high levels of parental criticism. A recent example is a pilot study which explored the integration of emotion‐coaching (EC) into standard FBT (Aarnio‐Peterson et al., 2024). This study focused on adolescents aged 12–17 years with AN or atypical AN, particularly in families exhibiting high levels of expressed emotion. Participants were assigned to either FBT + EC or FBT combined with a psychoeducational support group, highlighting the potential benefits of personalizing treatment strategies to address unique family dynamics. Both groups showed improvements in parental warmth and weight restoration, but the FBT + EC group demonstrated significantly higher parental warmth scores post‐treatment and were nine times more likely to achieve weight restoration at the three‐month follow‐up. These findings may suggest that integrating EC into FBT could be a valuable strategy for families with elevated expression of emotion, potentially improving treatment outcomes by fostering a more supportive and understanding family environment. While these results are promising, it is worth noting that most adjunctive interventions to FBT have so far yielded mixed findings. Further research is needed to clarify which adaptations yield the greatest benefit and for which families. Nevertheless, such innovative approaches mark an important move toward more personalized, flexible, and family‐responsive care in the treatment of adolescent AN.

However, advancing research on tailored interventions may require looking beyond large‐scale RCTs, as conducting such trials within the adolescent AN population presents significant challenges. Ethical concerns, medical fragility, and difficulties in sustaining long‐term participation often limit the feasibility of large trials, leading to high dropout rates and reduced statistical power (Lock et al. 2010). Given these barriers, an alternative and promising direction for future research is the use of (randomized) single‐case experimental designs (SCEDs). SCEDs offer a flexible and adaptive framework, making them particularly well‐suited for heterogeneous and high‐risk populations like adolescents with AN, where strict randomization and prolonged follow‐up are difficult to maintain. When rigorously designed, SCEDs can provide strong causal evidence by incorporating repeated measurements, systematic manipulation of interventions, and clearly defined baselines, making them a valid and powerful alternative to traditional RCTs for evaluating treatment effectiveness in this complex population (Vlaeyen et al. 2022). Nonetheless, the utility of SCEDs relies on careful design due to risks of confounding, measurement demands, and limited generalizability. In this context, meta‐analysis of SCEDs also holds promise for synthesizing findings across studies and enhancing generalizability (Maric et al. 2023).

In addition to SCEDs, other methodological innovations may also support progress beyond traditional RCTs. Improved routine outcome measurement across services and studies could enable the pooling of effect sizes from non‐randomized designs, thereby increasing the robustness and ecological validity of findings (Delgadillo et al. 2016). Furthermore, pragmatic trials offer a way to evaluate tailored interventions under real‐world clinical conditions, balancing scientific rigor with clinical relevance (Ford and Norrie 2016; Le‐Rademacher et al. 2023). In addition, innovations such as *synthetic control arms* derived from large electronic health‐record cohorts may help address feasibility and ethical challenges when randomization is difficult (Schaid and Lucchinetti 2023).

Lastly, it is important to recognize that current treatment guidelines recommending family therapy for adolescent AN are based almost exclusively on RCTs evaluating manualized FBT, a highly structured and specific form of intervention. While this evidence base supports FBT as a first‐line treatment, it does not automatically extend to broader or less clearly defined forms of family therapy. This distinction is critical, as non‐manualized or adapted approaches may differ in structure, delivery, and therapeutic mechanisms, and currently lack equivalent empirical support. Although research trials typically specify which treatment manual is being evaluated, treatment guidelines often refer more generally to “family therapy” without explicitly noting that their recommendations are grounded in studies of manualized FBT. Greater clarity in clinical guidelines is needed to prevent overgeneralization and ensure that implementation efforts align with the actual evidence. This would also support further research into which elements of family therapy are essential for treatment success and which adaptations may retain effectiveness.

## 5 Conclusion

The current review confirms the consistent and reliable effect of family‐based therapy approaches for adolescents with AN, primarily concerning somatic outcomes (specifically, weight restoration, remission, and reduced rehospitalization rates) across nine RCTs. However, evidence for sustained psychological recovery and long‐term outcomes remains limited and inconsistent. The overall evidence base is constrained by methodological limitations, including small sample sizes, heterogeneous designs, and short follow‐up periods. Consequently, larger and more rigorous trials are needed to determine long‐term treatment effects, identify mechanisms of change, and evaluate the added value of alternative or adjunctive interventions. Emerging personalized approaches, including tailored adaptations of family and individual therapy, warrant further investigation to clarify what works for whom and under which conditions these interventions best support both physical and psychological recovery. Sustained investment in high‐quality, collaborative research is therefore paramount to refine existing treatment guidelines and advance truly evidence‐based care for adolescents with AN.

## Author Contributions

**Renée A. Broersma:** conceptualization, methodology, investigation, data curation, writing – original draft and editing, visualization, project administration. **Moniek A. J. Zeegers:** conceptualization, methodology, investigation, data curation, writing – original draft and editing, visualization, project administration. **Fabienne Harteveld:** writing – review and editing. **Peer van der Helm:** writing – review and editing. **Mark Assink:** validation, formal analysis, writing – review and editing. **Jesse Roest:** coding effect sizes, validation, writing – review and editing. **Ramon J. L. Lindauer:** writing – review and editing. **James Lock:** writing – review and editing.

## Funding

The authors have nothing to report.

## Ethics Statement

Persons with lived experience of anorexia nervosa were consulted during the formulation of the review questions and to provide input on the relevance and patient‐centered interpretation of outcome domains. They also reviewed drafts of the manuscript to improve clarity and to ensure respectful, non‐stigmatizing language. Feedback was discussed by the author team and incorporated where feasible.

## Conflicts of Interest

James Lock reports being a co‐owner of the Training Institute for Child and Adolescent Eating Disorders. James Lock also reports receiving funding for clinical trials related to eating disorders and is an author of Family‐Based Treatment and Adolescent Focused Therapy, which are treatments discussed in this review. All other authors declare that they have no known competing financial interests or personal relationships that could have appeared to influence the work reported in this paper.

## Supporting information


**Data S1:** PRISMA 2020 checklist the effectiveness of psychological treatment for anorexia nervosa in adolescents.

**Appendix S1:** Search strategy.

## Data Availability

The data that support the findings of this study are available from the corresponding author upon reasonable request.
