# Supplementary material for: The Effectiveness of Psychological Treatment for Anorexia Nervosa in Adolescents: A Critical Review of Randomized Controlled Trials
Source: Int J Eat Disord. 2026 Jan 30;59(5):829–53. doi: 10.1002/eat.70031 (PMC13147141; doi:10.1002/eat.70031)
Supplement: Supplementary file 1 — Data S1: PRISMA 2020 checklist the effectiveness of psychological treatment for anorexia nervosa in adolescents. [file EAT-59-829-s002.docx]

## Supplementary material

### Appendix 1. Search strategy

Electronic databases were searched for relevant studies until June 2025 for articles, book chapters, dissertations, and reports on AN treatment in adolescents: Web of Science, APA PsycINFO, Google Scholar, Pubmed, Embase, Cochrane Library, and ERIC. The databases included Ovid MEDLINE (ALL <1946 to June 2025>), PubMed (to June 2025), APA PsycInfo (<1806 to June 2025>), Embase (<1974 to June 2025>), Cochrane Library (CENTRAL, CDSR), and Web of Science (Core Collection). The search strategy was tailored to each database to ensure the inclusion of relevant studies while minimizing irrelevant records.

The search terms covered three main categories: (1) Anorexia nervosa, (2) Treatment, and (3) Study design and population. The following search terms and their variations were used across all databases: "anorexia" OR "anorexia nervosa" (to capture the disorder), "treatment" OR "therapy" OR "intervention" (to include relevant therapeutic approaches), and "RCT" OR "randomized controlled trial" OR "comparison group" OR "placebo" (to focus on controlled experimental studies). To ensure the inclusion of adolescent-focused studies, we used terms such as "youth" OR "adolesc" OR "teenage" OR "child*"**.

Additional filters and exclusion criteria were applied to refine the results. Studies were limited to peer-reviewed journal articles. Cross-sectional studies were excluded using terms such as "NOT cross-sectional". To prevent the inclusion of studies unrelated to AN, we excluded research on cancer, diabetes, migraines, ADHD, and other non-psychiatric conditions (e.g., "NOT Cancer OR carcin* OR chemo* OR radiation OR migrain* OR leukemia OR cell* OR sugar OR diabet* OR bone OR ADHD"). Qualitative studies were removed using "NOT qualitative", and single-case or small-scale experimental designs were excluded with "NOT single-case OR single case OR SCED". Study protocols were removed with "NOT protocol".

The final search strategy was implemented separately for each database, with syntax adjustments to align with the specific search functionalities of each platform. Searches were performed without applying language filters. Consequently, no studies were excluded due to language during the screening process.
